# A hybrid statistical–machine learning framework for evaluating geomagnetic storm effects on MisrSat2 satellite power subsystems

**DOI:** 10.1038/s41598-025-22604-z

**Published:** 2025-10-29

**Authors:** Marwa S. Mostafa, Mohammed Abu Bakr Ali, N. Hesham, Yassin Mounir Yassin, Asmaa Ahmed, Dalia Elfiky

**Affiliations:** 1 National Authority for Remote Sensing and Space Science (NARSS), 23 Jozif Tito St., Cairo, 11769 Egypt; 2 Egyptian Space Agency (EgSA), Cairo, 1564 Egypt

**Keywords:** Geomagnetic storm, Satellite telemetry, Space weather impacts, Mixture of experts (MoE), Solar panel degradation, Climate sciences, Engineering, Mathematics and computing, Space physics

## Abstract

This study introduces a hybrid statistical–machine learning framework to evaluate the impact of the May 2024 geomagnetic storm on the power subsystem of the MisrSat-2 satellite. The proposed framework integrates a multi-tiered statistical approach, employing CUSUM for change point detection, z-score for outlier identification, and event-based analysis, with robust validation through Welch’s t-tests, bootstrapping, and Benjamini–Hochberg false discovery rate (BH-FDR) control. On 10 May, near the storm’s onset, the solar arrays showed modest current deviations, after validation, 13 events solar panel-1 and 17 events solar panel-2 were retained, with the largest cluster between 07:05 and 09:25 UTC. whereas the battery subsystem remained stable and buffered fluctuations, maintaining bus integrity. Event-based analysis confirmed that all deviations were small (< 4%) and within design tolerances. Radiation degradation modeling with EQUFLUX predicted only 0.32% cumulative loss for May 2024, align with the absence of measurable radiation-driven signatures in telemetry. Extending beyond descriptive detection, a Mixture of Experts (MoE) machine learning framework achieved superior predictive accuracy (R^2^ = 0.921, MAE = 0.063 A) compared to baseline models providing interpretable validation of statistical findings. The novelty of this research lies in its integrative approach, merging physics-based modelling, robust statistical methods, and interpretable machine learning to provide a scalable framework for anomaly diagnostics and mission assurance in dynamic space environments.

## Introduction

Satellites are indispensable cornerstones of modern technological infrastructure, facilitating everything from global communications and Earth observation to navigation and scientific research^1^. The continuous functionality and longevity of these spaceborne platforms critically depend on reliable telemetry, which serves as the vital link for monitoring the health, status, and performance of onboard subsystems^2^. Through meticulous analysis of telemetry data, mission operators can detect anomalies, implement timely corrective actions, and ensure uninterrupted mission continuity. As satellites grow in sophistication and assume increasingly vital roles, robust interpretation of telemetry under diverse and dynamic space environmental conditions becomes paramount for system reliability and resilience.

Among the myriad external factors influencing satellite operations, space weather presents a major source of uncertainty and potential disruption^3^. Events such as geomagnetic storms, Solar Energetic Particle (SEP) events, and fluctuations in the solar wind significantly alter the near-Earth space environment. These phenomena can induce detrimental effects, including surface charging, single event upsets, increased radiation doses, and variations in ionospheric conductivity. All these disturbances can perturb critical satellite subsystems, most notably power systems, and are directly reflected in observable telemetry behavior^4^. The solar panel subsystem, which directly interfaces with the external environment and is crucial for maintaining the satellite’s power balance, is particularly vulnerable to both transient and cumulative effects of space radiation.

The May 2024 geomagnetic storm stands out as a defining event of the current solar cycle (Solar Cycle 25), marking one of the most severe since Solar Cycle 24^5,6^. Driven by a massive coronal mass ejection (CME), this extreme storm plunged the Disturbance Storm Time (Dst) index below – 400 nT, categorizing it at a G5 level on the NOAA Geomagnetic Storm Scale—the highest classification. Accompanied by solar wind speeds exceeding 900 km/s and a sustained southward interplanetary magnetic field (IMF), the storm exerted strong coupling with Earth’s magnetosphere, with the K_p_ index reaching its maximum value of 9. The impacts were widespread, affecting ground-based technologies like power grids, disrupting high-frequency communications, and producing auroras at unusually low latitudes. For satellites in Low Earth Orbit (LEO), effects included increased atmospheric drag leading to orbital decay, enhanced radiation levels posing risks to electronics and solar panels^7^, and anomalies in power systems. For instance, past severe geomagnetic storms have caused the deorbiting of satellites^8^ and temporary operational failures^9^, underscoring the critical need for understanding such events.

Despite the widely recognized vulnerability of spacecraft to space weather, there remains a significant gap in empirical studies that investigate the direct, real-time influence of specific space weather events on satellite telemetry in operational contexts. Existing research has predominantly focused on long-term degradation models, laboratory-based testing, or broad correlations between solar indices and overall system health^10–12^. There is a critical need for extensive studies that integrate statistical techniques and advanced machine learning approaches to detect, characterize, and understand the influence of space weather on telemetry signals, particularly at high temporal resolution^12,13^ . Establishing a precise relationship between space weather events and satellite anomalies is crucial for developing proactive mitigation strategies.

Thus, this study addresses a critical gap in understanding the impact of extreme space weather on satellite performance by analyzing the MisrSat-2 satellite, launched in December 2023, during the May 2024 geomagnetic storm, focusing on its solar panel current and battery subsystem telemetry. MisrSat-2, a key asset in Egypt’s space program for Earth observation, lacks onboard sensors for detailed space weather anomaly detection, necessitating innovative analytical approaches. By integrating space weather parameters (e.g., solar wind speed, geomagnetic indices) with high-resolution telemetry, this work bridges space weather research and satellite anomaly analysis. A hybrid framework combining advanced statistical methods, a Mixture of Experts (MoE) machine learning model, and radiation damage simulation is employed to assess immediate and delayed storm effects on MisrSat-2’s performance, while enabling anomaly prediction and mitigation. The contributions of this paper are threefold.

1. Establishing a systematic approach for detecting and validating telemetry anomalies that coincide with geomagnetic disturbances.
2. Evaluating whether any observed changes in telemetry fall within design tolerances or are indicative of physical degradation.
3. Exploring the predictive power of ensemble learning models, particularly a Mixture of Experts (MoE) framework, in identifying space weather–induced anomalies. This includes benchmarking against established models like Linear Regression^14^ Random Forest^15^, XGBoost^16^, and Long Short-Term Memory (LSTM)^17^ neural networks^17^ to justify the MoE’s utility.

## Materials and methods

### Datasets

#### Space weather dataset

To investigate the influence of space weather on MisrSat-2’s power subsystem, a comprehensive set of solar and geomagnetic parameters was compiled. The space weather dataset used in this study was obtained from the Space Weather Data Portal^18^ and includes high-resolution measurements of both particle fluxes and geophysical indices. The dataset covers the following parameters: proton fluxes at energy thresholds of > 1 MeV (P1),  > 5 MeV (P5),  > 10 MeV (P10),  > 30 MeV (P30),  > 50 MeV (P50), and > 100 MeV (P100), measured in protons/cm^2^·s·sr; electron fluxes at > 0.8 MeV (E_8),  > 2.0 MeV (E2_0), and > 4.0 MeV (E4_0), measured in electrons/cm^2^·s·sr; solar wind parameters including proton density (particles/cm^3^), proton temperature (Kelvin), solar wind density (particles/cm^3^), solar wind speed (km/s), and solar wind temperature (K); geomagnetic indices such as AE, AL, AU, SYM-D, SYM-H, ASY-D, and ASY-H; and the three components of the interplanetary magnetic field in GSE coordinates (BGSEc_1, BGSEc_2, BGSEc_3, in nT).

The selected period spans from May 1 to May 31, 2024, during which an extreme geomagnetic storm was recorded. The compiled dataset includes 8928 entries across 28 features, forming a high-quality, structured basis for correlation and anomaly analysis with satellite telemetry.

All variables were resampled to 5 min intervals to match the temporal resolution of the satellite telemetry data. Prior to analysis, all variables were normalized to ensure consistency across feature scales. Notably, the dataset was complete and free from missing values or gaps, which enhanced the reliability of the subsequent analysis and model training.

#### MisrSat-2 telemetry data

MisrSat-2 is equipped with two identical solar array wings, each comprising a single panel with dimensions of 1400 mm by 1000 mm, yielding a total exposed area of 2.8 m^2^. The panels use triple-junction GaInP₂/GaAs/Ge solar cells, bonded to the panel surface with space-qualified adhesives and protected by radiation-resistant cover glasses. These cells, designed to operate under standard space conditions (135.3 mW/cm^2^ at 25 °C), offer a high conversion efficiency of 31%. The solar array is directly exposed to the space environment, so both radiation shielding and thermal control were carefully implemented during the design phase to ensure long-term reliability over the satellite’s 5.25 year mission lifetime. This configuration ensures high electrical performance while withstanding radiation-induced degradation and thermal fluctuations in low Earth orbit.

Telemetry data from MisrSat-2 were provided by the Egyptian Space Agency (EgSA), covering the same observation period from May 1 to May 31, 2024. The analysis focused on key electrical parameters of the power subsystem, including Battery voltage (volt_battery), Battery current (curr_battery), Current from Solar Panel 1 (curr1_solar_panel) and Current from Solar Panel 2 (curr2_solar_panel).

The telemetry was initially recorded at 1 s intervals. However, to ensure alignment with the space weather dataset and to smooth high-frequency noise, the data were resampled to a 5 min resolution. This preprocessing step preserved significant trends, thereby enabling more robust comparison and model training.

Approximately 10% of the telemetry data contained missing values, which were addressed during the data preprocessing stage using standard imputation techniques (details provided in Sect. “Orbit modeling and illumination flag generation”). Baseline values observed for the power subsystem were as follows: Battery voltage: ~ 27–29 V, Battery current: ~ 10–13 A, Solar panel currents: ~ 10–13 A for each panel. These operational values serve as the reference state for identifying deviations potentially associated with geomagnetic disturbances.

### Proposed workflow

**Fig. 1:**
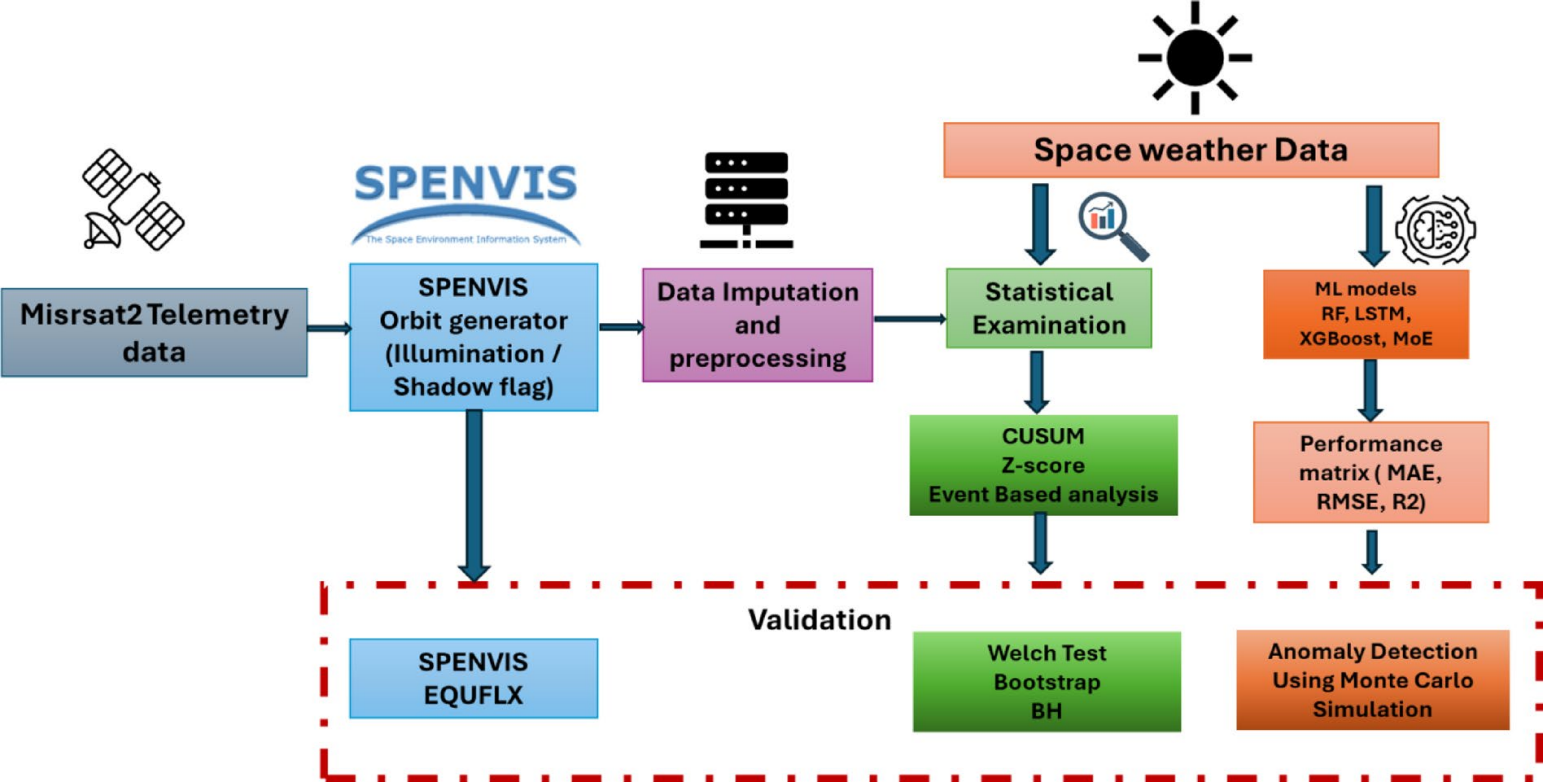
The proposed framework for detecting the effects of space weather on MisrSat2 solar panels.

Figure 1 illustrates the hybrid statistical–machine learning framework used to evaluate the impact of extreme space weather events specifically the May 2024 geomagnetic storm, on the MisrSat-2 satellite’s telemetry data, with a focus on its solar panel current and battery subsystem. The framework initiates with the collection of MisrSat-2 telemetry data and concurrent space weather parameters, including solar wind speed, density, and geomagnetic indices. Using the satellite’s orbital parameters, the SPENVIS Orbit Generator^19^ was employed to determine illumination and shadow periods experienced by the solar panels, enabling the correlation of sunlight exposure with solar array current. This illumination/shadow information was incorporated into the preprocessing stage, which involved filling missing values, applying interpolation, and resampling telemetry data to a 5 min resolution to ensure temporal alignment with space weather datasets.

The statistical examination stage applies three lenses—CUSUM, z-score, and event-based analysis—to screen for storm-time deviations (CUSUM targets small mean shifts with calibrated average-run-length behaviour). Detected candidates then enter a validation layer (Welch *t*-tests, bootstrap resampling, and Benjamini–Hochberg FDR control) to retain only statistically robust events.

To quantify the physical degradation of the solar panels, the EQUFLUX model in SPENVIS was used to estimate radiation-induced efficiency loss for the specific panel type. These simulated degradation levels use as a qualitative benchmark for the level of radiation-driven change expected given the environment.

In the ML branch, multiple predictors (RF, LSTM, XGBoost, and MoE) are trained to forecast telemetry from space-weather inputs; performance is summarized with MAE, RMSE, and R^2^, and Monte-Carlo residual analysis is used for probabilistic anomaly detection. The two branches—statistics + benchmarking (with EQFLUX context) and ML + residual anomalies—converge to an integrated interpretation of storm impacts on the solar arrays and the regulated battery bus.

#### Orbit modeling and illumination flag generation

To characterize illumination conditions during the analysis period, we used the SPENVIS Orbit Generator (v4.6.12.3517) to simulate MisrSat-2’s orbit over May 2024, based on its actual orbital parameters (629 km altitude, 97.5° inclination, ~ 90 min period). The simulation provided eclipse intervals for each orbit, enabling the creation of a binary Sunlight/Shadow flag (1 for sunlight, 0 for eclipse). These flags were interpolated across the telemetry time series, allowing precise labelling of data points with respect to solar exposure, critical for isolating power variations due to orbital geometry from those driven by space weather effects.

#### Data imputation and preprocessing

Prior to event-based analysis and machine learning modelling, MisrSat-2 telemetry underwent preprocessing with emphasis on illumination state. A binary Sunlight/Shadow flag, generated via SPENVIS orbit simulation, guided imputation and normalization. Missing values in solar panel and battery telemetry were imputed using a flag-aware strategy: time-based linear interpolation during sunlight (flag = 1) preserved smooth power trends, while conservative methods (backward fill or nearest-neighbour imputation) were applied during eclipse (flag = 0) to maintain physical consistency. For mixed-flag gaps, interpolation respected flag boundaries. All channels were resampled to uniform 5 min intervals, aligning with space weather data and ensuring physically realistic inputs for downstream analysis of the May 2024 geomagnetic storm effects.

#### Statistical change point and outlier detection

To assess the impact of the May 2024 geomagnetic storm on MisrSat-2’s power subsystem, we developed a statistical framework comprising three complementary methods to detect, validate, and quantify anomalies in solar panel currents (curr1_solar_panel, curr2_solar_panel) and battery parameters (volt_battery, curr_battery). These methods—Cumulative Sum (CUSUM) for change points, z-score for outliers, and event-based analysis for storm-specific assessment—enable robust identification of storm-related shifts while distinguishing them from routine operational variations. All detections undergo multi-tiered validation to address small sample sizes and multiple comparisons.

*The Cumulative Sum (CUSUM)*^20^ was applied to monitor mean shifts in solar panel and battery current data, potentially caused by environmental disturbances such as geomagnetic storms. The algorithm computes cumulative statistics using Eqs. (1 and  2):

1 $${\text{S}}_{{\text{t}}}^{ + } = \, \max \left( {0, \, S_{{{\text{t}} - {1}}}^{ + } \, + \, \left( {x_{{\text{t}}} \, - \, \mu } \right)/\sigma \, - {\text{ k}}} \right)$$

2 $${\text{S}}_{{\text{t}}}^{ - } \, = {\text{ min}}\left( {0,{\text{ S}}_{{{\text{t}} - 1}}^{ - } \, + \, \left( {{\text{x}}_{{\text{t}}} \, - \, \mu } \right)/\sigma \, + {\text{ k}}} \right)$$

A change point is detected when the cumulative sum exceeds a threshold |S⁺ₜ|> k or |S⁻ₜ|> k). To avoid redundant detections, points within a 15 min window are merged, retaining only the earliest. In the operational script, a single parameter (threshold = 2) was used for both k and h, a conservative plan that prioritizes robustness against false positives. To estimate the expected false alarm rate (FAR) under the adopted CUSUM settings, a Monte-Carlo Average Run Length (ARL) simulator was run on standardized in-control data. From ARL₀, the FAR per sample was computed as FAR = 1/ARL₀, allowing comparison of observed detections with the theoretical false alarm expectation.

*The z-score Outlier Detection* To identify localized deviations, z-scores were calculated for each time point

3 $${\text{z}}_{{\text{t}}} \, = \, \left( {{\text{x}}_{{\text{t}}} \, - \, \mu } \right) \, / \, \sigma$$

with points exceeding |zₜ|> 2, flagged as outliers (~ 95% coverage under normality assumptions). These outliers, which may reflect transient effects like illumination shifts or space weather disturbances, were cross-checked with CUSUM change points to differentiate true anomalies from statistical noise^21^^,^^22^.

*Event-Based Statistical Analysis for Storm Impact Detection* As a third method to detect and contextualize anomalies, we performed an event-based analysis over an 8-day symmetric window (four days before and after May 11, 2024), focusing on sunlight periods to isolate space weather effects from orbital dynamics. This approach complements CUSUM and z-score by quantifying storm-specific changes in telemetry.

*Significance Testing* The Wilcoxon signed-rank test, a non-parametric method suitable for paired samples, evaluated differences between pre-and post-storm telemetry (*p* < 0.05 indicating significance). Stratifying data by illumination states minimized distortion from non-normal distributions and orbital effects.

*Effect Size Quantification* To measure the magnitude of detected changes, we calculated matched-pairs Cohen’s d for each variable (d = (X̅_post − X̅_pre)/s_d, where s_d is the standard deviation of paired differences^25^). Non-parametric bootstrapping (10,000 resamples) provided 95% CIs for Cohen’s d, with CIs excluding zero indicating reliable effects^23^.

#### Validation of detected anomalies

To ensure the statistical robustness of anomalies identified across all three methods, we implemented a multi-tiered validation approach,

*Welch’s T-Test (for CUSUM Change Points)* For each CUSUM-detected change point, we compared telemetry means in 15 min windows (three 5 min observations per side) before and after the event using two-sample Welch’s t-tests, which accommodate unequal variances. Change points with *p* < 0.05 were deemed significant, indicating likely physical or environmental drivers^24^.

*Bootstrapping Enhancements* For each detected change point, we compared adjacent “before” and “after” windows (n = 3 per side) using a nonparametric percentile bootstrap of the mean difference ${\Delta } = {\text{after}} - {\text{before}}$. We generated 10,000 paired resamples by sampling with replacement within each window, recomputed ${\Delta }^{*}$ for each resample, and formed a 95% percentile confidence interval from the empirical ${\Delta }^{*}$ distribution. An effect was considered supported when the 95% CI excluded zero. This approach is distribution-free and well suited to small-sample windows, providing a robust check on the detected shifts without assuming normality^25^.

*False Discovery Rate (FDR) Control* To manage multiple comparisons across ~ 29–30 change points per solar panel, we applied the Benjamini-Hochberg (BH) procedure at q = 0.05^24^. *P* values were sorted (p₁ ≤ … ≤ pₘ), and significance was declared for the largest k where ${p}_{(k)}\le \frac{k}{m}q$. Equivalently, BH q-values were computed as ${q}_{(i)}={min}_{j\ge i}\{\frac{m}{j}{p}_{(j)}$(capped at 1), with anomalies considered significant when q ≤ 0.05 and bootstrap CIs excluded zero. This approach minimized false discoveries, even under dependence, and was applied uniformly to validated outputs from all three detection methods^26^.

#### Machine learning model for telemetry prediction

The Mixture of Experts (MoE) combines multiple specialized sub-models via a router network^27^. Operating on a divide-and-conquer principle, the router network divides complex problems into subspaces, assigning each to a corresponding expert for solution integration^28^^,^^29^. Each expert specializes in a specific data subset or feature, enhancing its ability to capture distinct patterns^30^. This approach allows the model to dynamically adjust expert weights, blending predictions to boost performance and robustness. Compared to a single ML model, MoE offers superior generalization and efficiently balances performance with computational cost. As deep learning models grow in size and resource demands, MoE has become a key focus in large-scale model research, yielding improved accuracy.

**Fig. 2:**
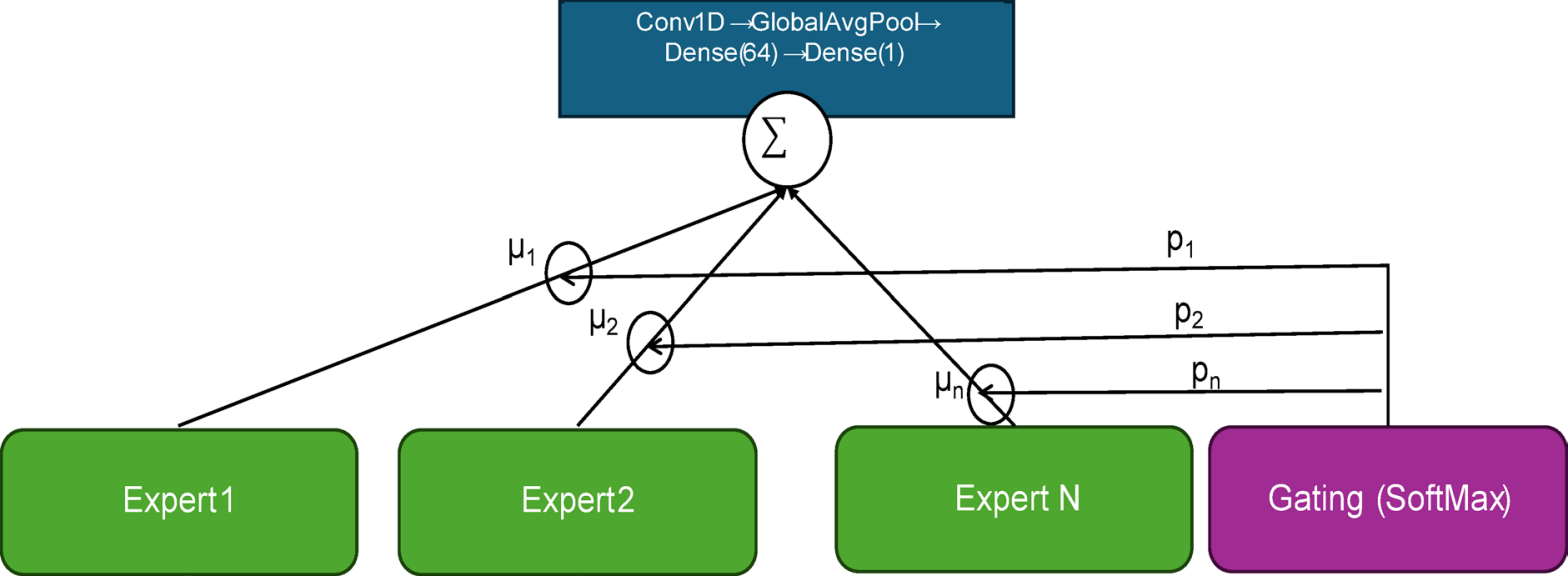
Schematic overview of MoE-AE.

In this section, we describe the architecture of the proposed MOE model employed to predict the maximum output current (`max_curr`) of a solar panel, shown in Fig. 2. To model the non-linear dynamics of solar panel output under varying illumination and shadow conditions, we incorporate Mixture-of-Experts (MoE) layers into our forecasting models. Each MoE layer consists of n expert subnetworks E_1_, E_2_, …, E_n_ and a gating network G, which outputs a probability distribution p_1_, p_2_, …, p_n_ for selecting each expert. The gating mechanism dynamically routes each input. This allows the model to adapt to distinct operational conditions. In the soft-gating mechanism, the gate output is computed in Eq. (4).

4 $$G\left(x\right)= Softmax\left(x . Wg\right)$$

where x is the input feature vector, and $Wg$ is a trainable weight matrix. This ensures that each input activates all experts proportionally, enabling robust learning across diverse illumination and shadowing scenarios. Table 1 illustrated the proposed architecture in detail. The gating outputs of the MoE model were used to identify the dominant expert at each timestep. Expert activation patterns were visualized through heatmaps and spider plots summarizing mean standardized feature values per expert. These tools enhance interpretability by linking physical conditions to expert selection, enabling a semi-physical understanding of model behaviour.

**Table 1:** Expert network architecture (for each expert) and gating network architecture.

Layer type	Configuration	Output shape
*Gating network architecture*		
Input	Input sequence of shape (24, 24)—24 time steps × 24 features	(batch_size, 24, 24)
LSTM	64 units, return_sequences = False	(batch_size, 64)
Dense	5 units (number of experts), activation = ‘softmax’	(batch_size, 5)
*Expert network architecture*		
Conv1D	32 filters, kernel_size = 3, activation = ‘relu’, padding = ‘same’	(batch_size, 24, 32)
GlobalAveragePooling1D	Aggregate features across time dimension	(batch_size, 32)
Dense	64 units, activation = ‘relu’	(batch_size, 64)
Dense	1 unit	(batch_size, 1)

A key challenge in MoE architectures is uneven expert usage, where a few experts dominate predictions while others remain underutilized (the *dying experts* problem). This can reduce generalization, particularly when predicting max_curr across varying conditions. We compute the Expert Importance (EI) as the sum of gate values across a training batch

5 $$EI\left(x\right)=\sum_{x\in X}G\left(x\right)$$

To encourage balanced usage, we penalize the coefficient of variation (CV) of expert importance.

6 $${EI}_{loss}=wei\cdot {\left(\frac{\sigma \left(EI\right)}{\mu \left(EI\right)}\right)}^{2}$$

This regularization ensures that all experts contribute to the predicting especially for diverse input patterns. Additionally**,** to ensure experts learn distinct representations of solar panel behaviour, we introduce orthogonality penalties on both expert outputs and weight vectors. The normalize expert outputs EO using the L2 norm.

7 $${E}_{norm}=\frac{EO}{\sqrt{\mathbf{m}\mathbf{a}\mathbf{x}\left(\sum {EO}^{2},\varepsilon \right)}}$$

Compute the output diversity loss.

8 $$EO_{loss} = weo \cdot \mathop \sum \limits_{x \in X} \left( {E_{norm} \cdot E_{norm}^{T} - I} \right)^{2}$$

A similar penalty, ${EW}_{loss}$ is applied to expert weight vectors to encourage structural diversity in parameter space, ensuring experts specialize in distinct aspects of the data.

**Loss function**

The combined loss function for training is

9 $$Loss = RMSE + w \cdot \left( {EL_{loss} + EO_{loss} + EW_{loss} } \right)$$

where RMSE measures the prediction error for max_curr, and $w$ is a decay factor reduced over epochs to allow adaptive imbalance as training converges. This loss function balances prediction accuracy with expert utilization, critical for handling the dataset’s variability. The factor $w$ was initializes at 0.1 and decays exponentially as ${w}_{t}=0.1\times {0.95}^{t}$, where t denotes the epoch, promoting stable routing convergence.

#### Anomaly detection via probabilistic residual simulation

To detect anomalies in solar panel current, a probabilistic approach based on Monte Carlo simulation of residuals was employed. For each timestep t, the residual r_t_ is defined as the difference between the actual observed value y_t_ and the model’s prediction $y_{t}^{ \wedge }$ from the best-performing model (MoE):

10 $$r_{t} = y_{t} - y_{t}^{ \wedge }$$

A Monte Carlo simulation was then conducted by generating synthetic error distributions. This involved resampling with replacement from the observed residuals {r_1_,r_2_,…,r_N_} to create a large number of bootstrapped residual sets^31^^,^^25^. From these simulated residual distributions, 90% prediction intervals were estimated for future predictions. A prediction interval for a new observation y_new_ at timestep t can be constructed around the model’s prediction $y_{t}^{ \wedge }$ by considering the distribution of the residuals. Specifically, the lower bound (L_t_) and upper bound (U_t_) of the prediction interval are estimated as:

11 $${\text{L}}_{{\text{t}}} = {\text{y}}_{{\text{t}}}^{ \wedge } + {\text{Q}}_{0.05} ({\text{simulated}}\;{\text{residuals}})$$

12 $${\text{U}}_{{\text{t}}} = {\text{y}}_{{\text{t}}}^{ \wedge } + {\text{Q}}_{0.95} ({\text{simulated}}\;{\text{residuals}})$$

where Q_0.05_ and Q_0.95_ represent the 5th and 95th percentiles of the bootstrapped residual distribution, respectively^32^. Anomalies were subsequently flagged when true measurements y_t_ fell outside these statistically derived bounds (y_t_ < L_t_ or y_t_ > U_t_), indicating a significant deviation from expected behavior^20^. Daily anomaly counts were then computed and compared to known storm periods to validate their alignment with external events, further confirming the efficacy of the detection method.

## Results and discussion

This section presents the findings from our hybrid statistical–machine learning framework, structured to trace the impact of the May 2024 geomagnetic storm on MisrSat-2’s power subsystem. We begin by characterizing the storm’s space weather environment, followed by the satellite’s orbital context, statistical detection of outlier, Robust validations (e.g., Welch’s t-tests, bootstrapping, Benjamini–Hochberg procedure, Cohen’s d with confidence intervals) and performance metrics strengthen our findings. The machine learning model incorporates space weather parameters (e.g., proton fluxes, solar wind properties, geomagnetic indices) to investigate the causes of current spikes identified as statistical anomalies, complemented by event-based analysis and probabilistic anomaly detection. Performance metrics further strengthen our findings. An integrated discussion synthesizes the results, emphasizing system resilience and the framework’s novelty for space weather impact assessment.

### Temporal evolution of space weather parameters highlighting the geomagnetic storm

**Fig. 3:**
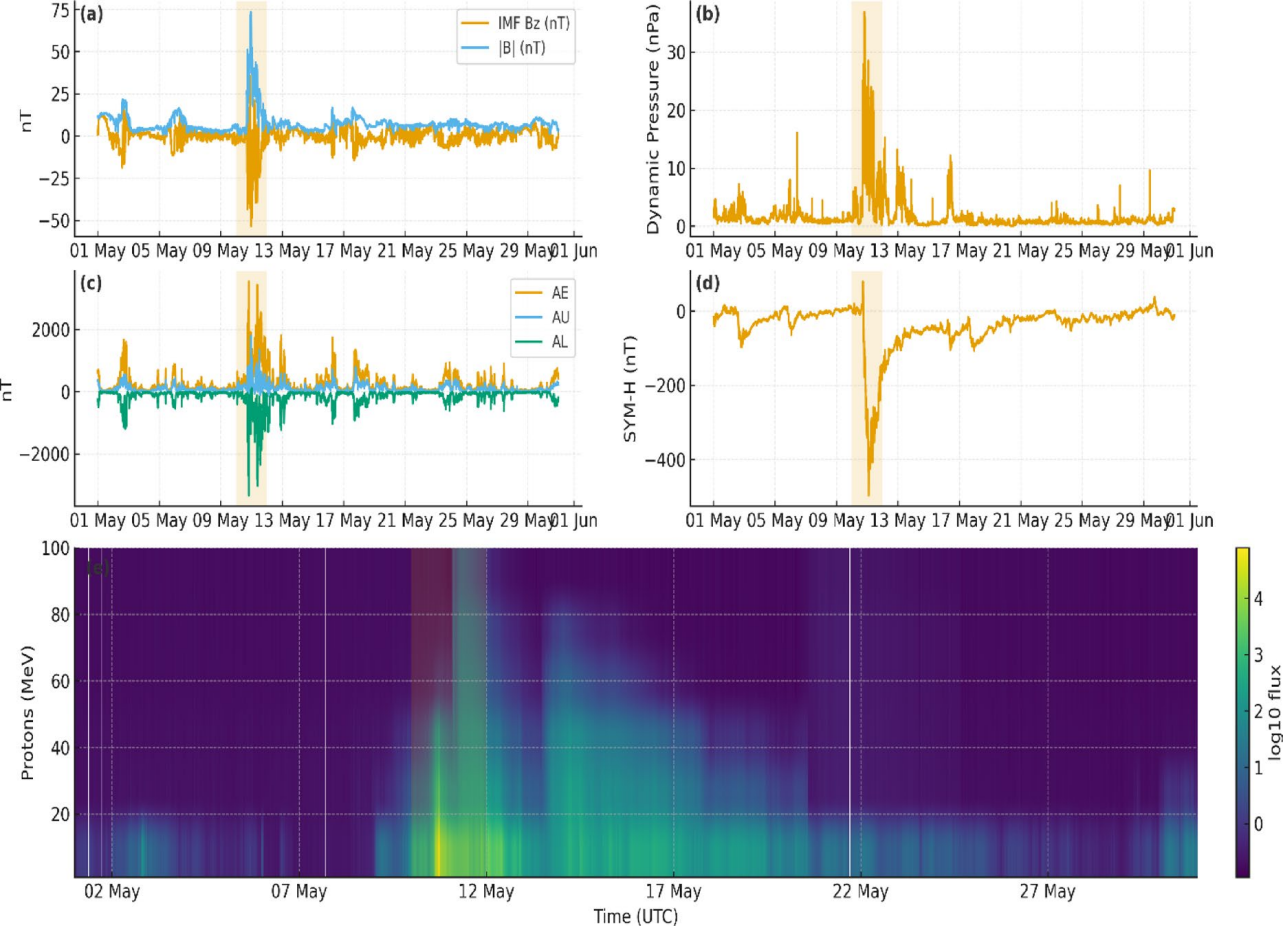
Space-weather conditions in May 2024 (5-min cadence). Shaded band marks the 10–11 May storm. Panels: (**a**) IMF ${B}_{z}$ and $\mid B\mid$, (**b**) solar-wind dynamic pressure ${D}_{p}$, (**c**) AE/AU/AL, (**d**) SYM-H, (**e**) proton flux spectrogram (${log}_{10}$). Time axis: day–month (UTC).

Figure 3 illustrates the time-series evolution of key space weather parameters from 1 to 31 May 2024, with emphasis on the geomagnetic storm that occurred on 11 May. The dataset includes proton fluxes (P1–P100), solar wind properties (proton density, temperature, solar wind speed, density, and temperature), geomagnetic indices (AE, AL, AU, SYM-H, SYM-D, ASY-H, ASY-D), and interplanetary magnetic field (IMF) components (BGSEc_1–3). The storm is clearly marked by a sharp decrease in SYM-H and SYM-D, indicating ring current intensification, and a corresponding increase in AE and AL indices, reflecting strong auroral activity. These changes coincide with an abrupt rise in solar wind speed and density and a pronounced southward turning of the IMF, particularly in BGSEc_1, the typical signatures of a geoeffective coronal mass ejection (CME) or interplanetary shock. Proton and electron fluxes also show marked enhancements, consistent with solar energetic particle (SEP) injections and magnetospheric particle acceleration.

Temporal offsets are evident across several parameters: for instance, solar wind and particle enhancements precede the minimum in SYM-H, while geomagnetic indices respond more abruptly. These lags must be considered when analyzing cause-effect relationships, especially in satellite impact studies. This event provides a critical case for studying the effects of space weather on satellite solar panels. Proton enhancements, particularly in the 1–30 MeV range, can cause displacement damage and reduce solar cell efficiency. Increased relativistic electron fluxes contribute to surface charging and transient power anomalies^33^. Rapid magnetospheric compression may also expose the satellite to harsher radiation regions and shift operational conditions such as attitude and thermal balance, indirectly affecting power generation. With this extreme storm environment established, we examine how MisrSat-2 telemetry responded.

### Orbit modeling and illumination flag generation

**Fig. 4:**
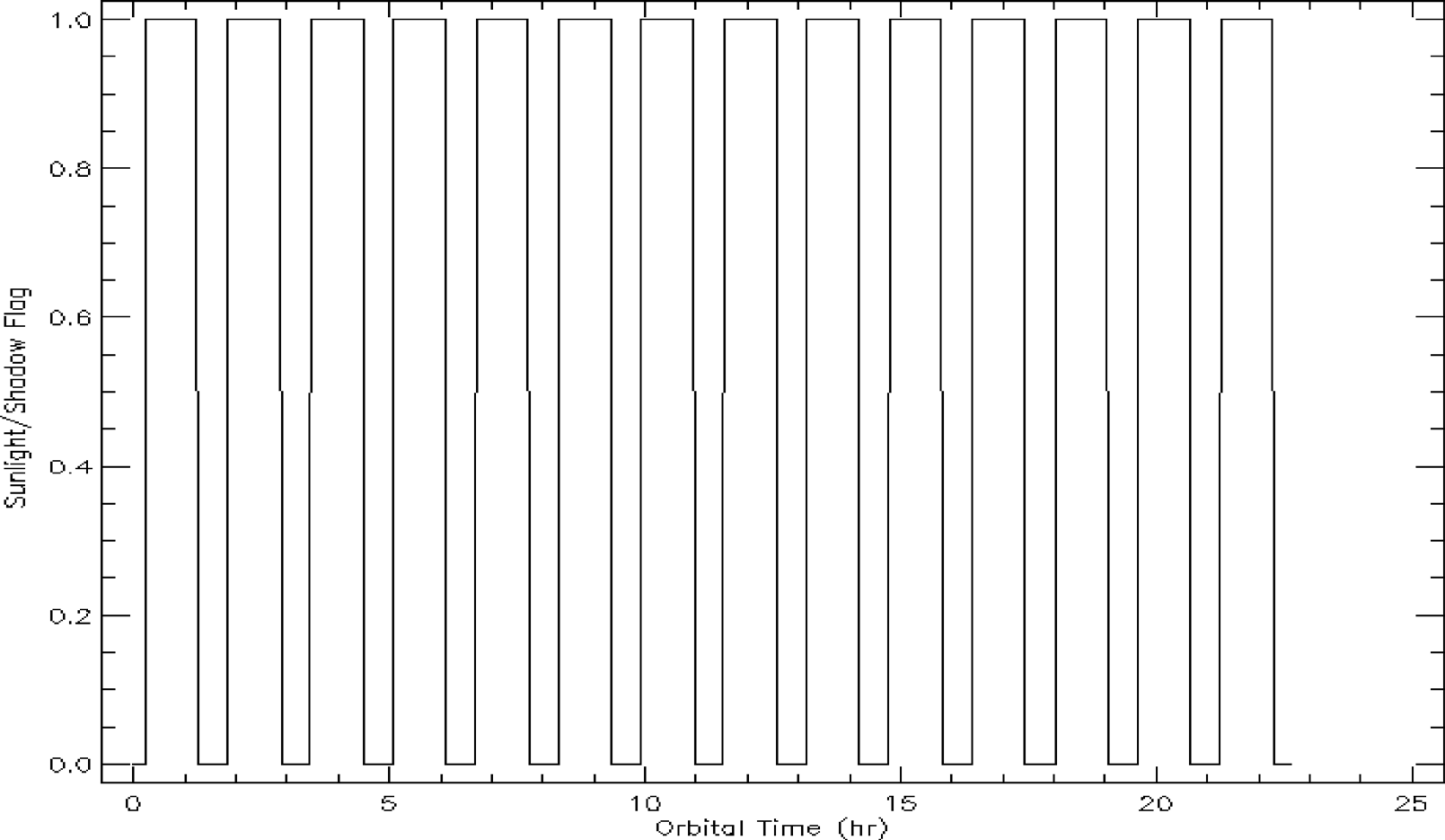
Binary sunlight-shadow flag profile over a 24 h orbital period. The value 1 indicates that the satellite is in direct sunlight, while 0 denotes passage through Earth’s shadow (eclipse). Each cycle represents one orbital revolution, illustrating the periodic alternation between sunlight and shadow typical for a Sun-synchronous low Earth orbit (LEO).

To isolate storm-driven effects from natural orbital variability, telemetry was segmented into sunlight (flag = 1) and eclipse (flag = 0) using orbit modeling Fig. 4. Each ~ 90 min orbit consists of ~ 60 min illumination and ~ 30 min eclipse, producing predictable oscillations in solar panel currents. This illumination segmentation provides a baseline to distinguish normal cyclic behaviour from storm-induced anomalies.

### Statistical change point and outlier detection: first-level anomaly screening

To investigate whether the May 2024 geomagnetic storm induced detectable anomalies in MisrSat-2 telemetry, we applied three complementary statistical methods: (i) CUSUM change point detection, (ii) z-score outlier analysis, and (iii) event-based analysis. Each method provides a different perspective on anomaly behaviour: CUSUM tracks gradual shifts in the mean, z-scores flag sharp point deviations, and event-based analysis contextualizes changes within the storm window. Together, these approaches form a layered screening strategy to ensure that storm-driven effects are not mistaken for normal orbital variability.

#### CUSUM change point detection

The Cumulative Sum (CUSUM) method was applied to MisrSat-2 telemetry to detect step-like changes in mean values potentially linked to the May 2024 geomagnetic storm. Analysis was performed on illumination-only data between May 9–15, 2024, resampled to 5 min cadence. To ensure that these detections and non-detections reflected true system behaviour rather than statistical artifacts, we calibrated the CUSUM settings (k = h = 2) using a Monte Carlo Average Run Length (ARL) simulator. The in-control ARL₀ was ≈ 6,760 samples, corresponding to a very low false-alarm rate (~ 0.015% per sample, or about one false alarm every 23–24 days at 5 min cadence). For a 1σ mean shift, the out-of-control ARL₁ was ≈251 samples, indicating a well-calibrated detector that responds quickly to genuine shifts while rejecting noise.

**Fig. 5:**
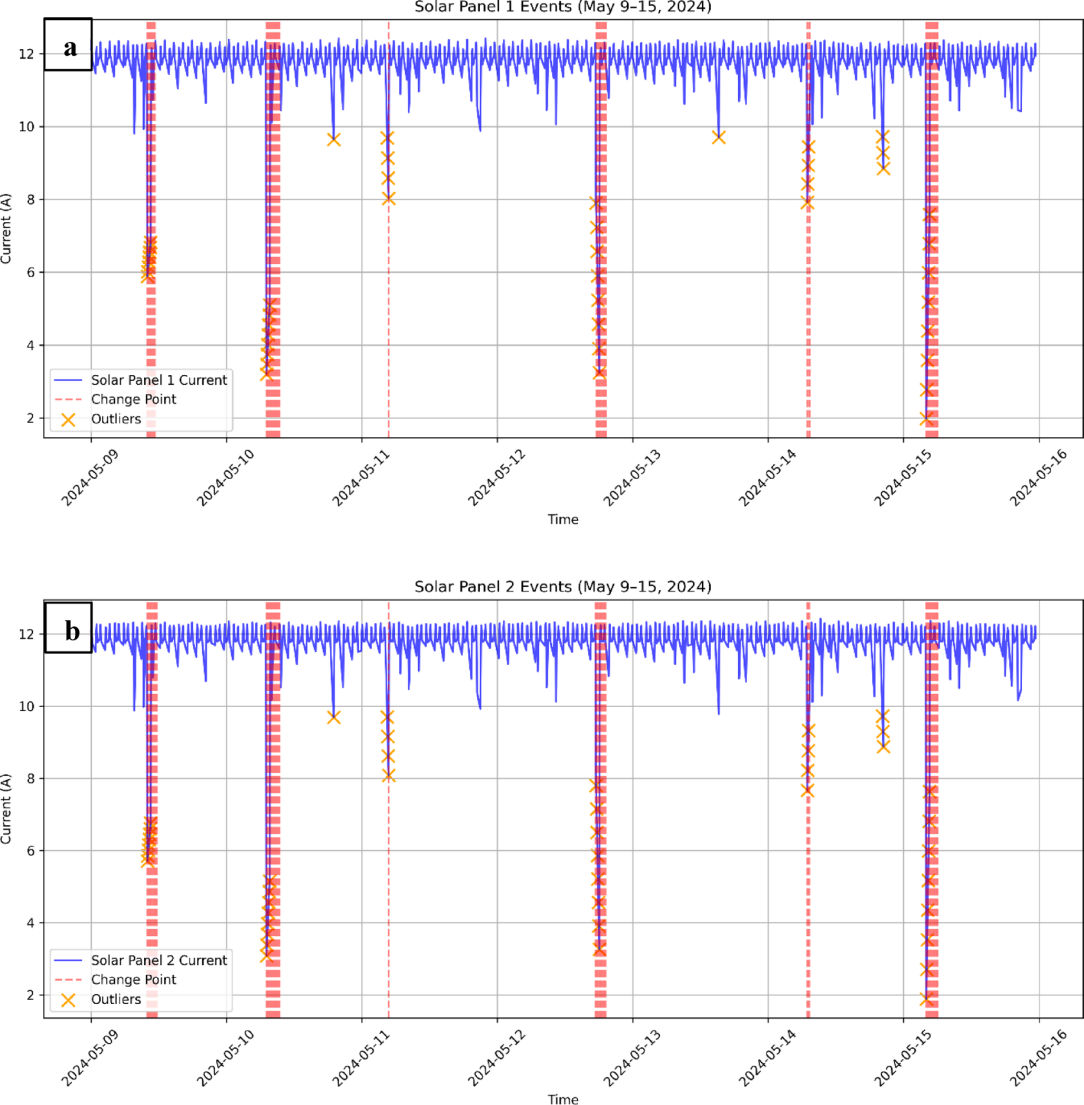
The detected events in (**a**) solar panel 1 and (**b**) solar panel 2.

**Fig. 6:**
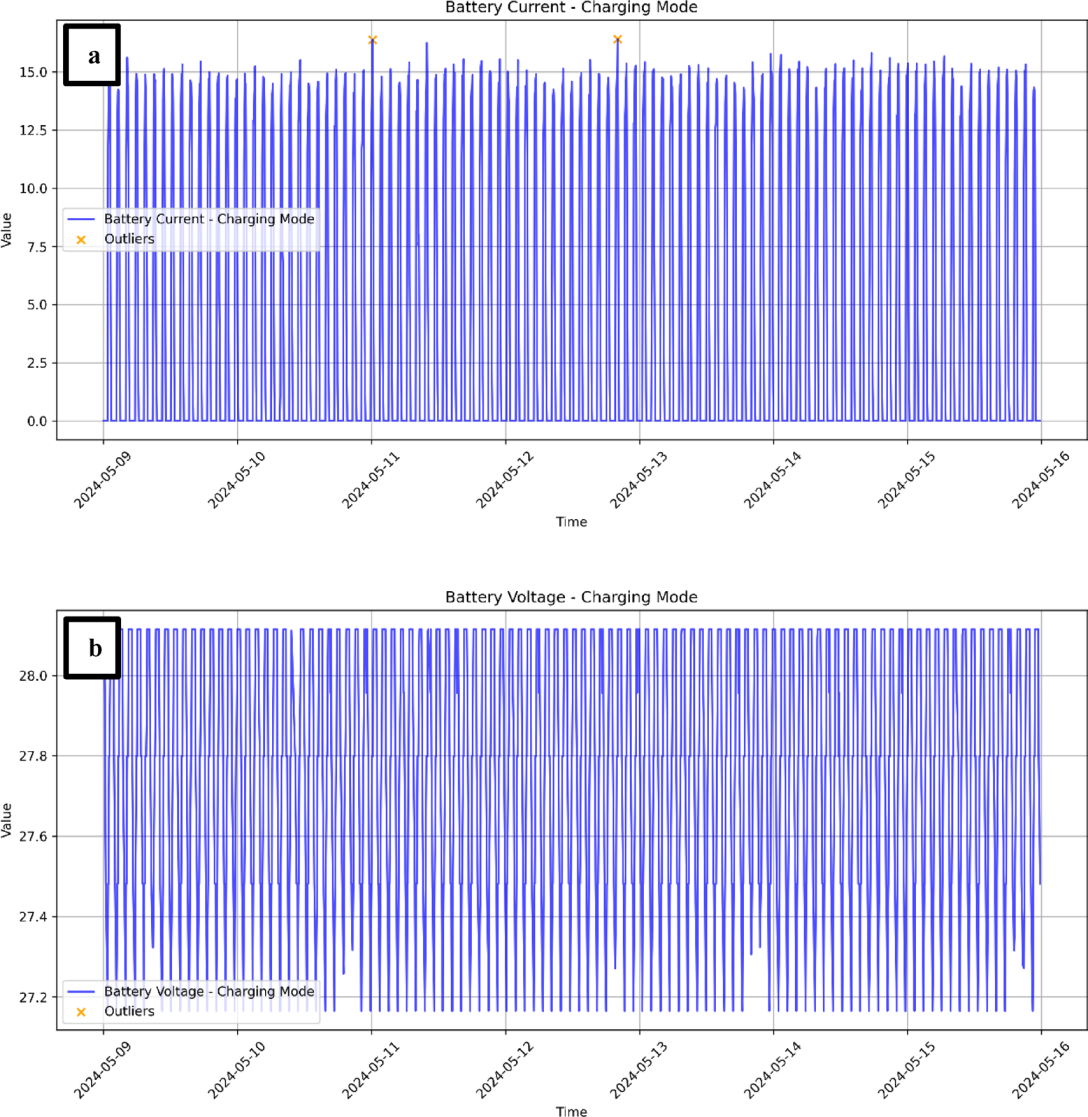
(**a**) Battery current in charging mode (May 9–15, 2024), (**b**) battery voltage in charging mode (May 9–15, 2024).

For the solar panels, CUSUM identified 29 candidate change points in Solar Panel-1(SP1) and 30 in Solar Panel-2 (SP2). Of these, 14 (SP1) and 16 (SP2) were statistically significant (*p* < 0.05, Welch’s t-test; Fig. 5a, b). Confirmed detections clustered strongly on May 10, aligned with the storm onset. In contrast, May 11, the peak disturbance day in geomagnetic indices, produced fewer detections, with no large robust clusters across both panels (Fig. 6).

Extending the analysis to the battery subsystem revealed a markedly different picture. Battery current oscillated regularly between 0 and 15 A, reflecting the expected orbital charging–discharging cycle, while battery voltage remained tightly regulated between 27.2 and 28.1 V (Fig. 6 a, b). Although CUSUM flagged a few candidate anomalies (e.g., current spikes above 15.5 A), none were validated by Welch’s t-test (*p* > 0.05). Thus, no sustained or abrupt shifts were detected in either current or voltage, confirming the robust stability of the battery system during the storm.

This contrast between subsystems is instructive: whereas the solar panels responded directly to the disturbed space environment with clusters of significant change points on May 10, the battery system acted as a buffer, absorbing fluctuations and preserving stable voltage output. This buffering behaviour highlights the robustness of MisrSat-2’s power regulation design, ensuring that external disturbances at the array level did not propagate into the spacecraft’s regulated power bus.

#### Z-score outlier detection

Whereas CUSUM highlights persistent step-like changes, some disturbances may appear only as short-lived excursions in the telemetry. To capture these transient behaviors, we applied a z-score analysis, flagging any point exceeding |z|> 2 as an outlier.

For the solar panel currents, z-score detection revealed a series of sharp spikes distributed across the May 9–15 window. Many of these spikes coincided with previously identified CUSUM change points, which strengthens confidence that those events represent genuine anomalies rather than gradual drift. A smaller fraction of outliers occurred independently, often near transitions between sunlight and eclipse. These isolated points are best interpreted as operational effects of orbital cycling rather than space weather signatures.

For the battery subsystem, z-score detection identified a limited number of statistically significant deviations. Battery voltage remained within its regulated range and did not exceed the threshold, while a few current spikes were observed but failed subsequent statistical validation. This pattern is consistent with the overall stability of the battery seen in CUSUM analysis and reinforces its role as a regulator of short-term fluctuations.

By confirming overlap with CUSUM detections and isolating spurious transitions, the z-score method provided an additional layer of anomaly screening. Together, CUSUM and z-scores establish that MisrSat-2 experienced both step-like shifts and transient spikes in solar panel currents during the storm period. However, these methods remain point-focused; they cannot determine whether the storm produced broader systematic changes in telemetry averages. To address this limitation, we next performed an event-based analysis over multi-day windows centered on the storm.

#### Event-based analysis

While CUSUM and z-score methods are effective for identifying point-level changes, they are inherently local and do not capture whether the geomagnetic storm produced sustained shifts in telemetry behaviour across multiple days. To address this, we implemented an event-based analysis, comparing pre- and post-storm averages within symmetric windows centered on May 11, 2024. Window lengths of ± 2, ± 3, and ± 4 days were tested to evaluate the sensitivity of results to the chosen time span. Only illumination periods were included to minimize orbital effects. The effect sizes, quantified using matched-pairs Cohen’s *d*, were consistently small but reliable (≈0.09–0.11), with bootstrapped 95% confidence intervals excluding zero as presented in Table 2.

**Table 2:** Effect sizes are matched-pairs Cohen’s d with bootstrapped 95% CIs.

Telemetry channel	± 2 days (Cohen’s *d*, 95% CI)	± 3 days (Cohen’s *d*, 95% CI)	± 4 days (Cohen’s *d*, 95% CI)
Solar Panel 1 current	0.10 (0.05–0.15)	0.10 (0.06–0.14)	0.11 (0.07–0.15)
Solar Panel 2 current	0.09 (0.04–0.14)	0.10 (0.06–0.14)	0.10 (0.06–0.14)
Battery voltage	0.06 (0.02–0.10)	0.09 (0.05–0.13)	0.12 (0.08–0.16)
Battery current	0.07 (0.01–0.12)	0.03 (–0.01–0.07)	0.002 (–0.03–0.03)

**Fig. 7:**
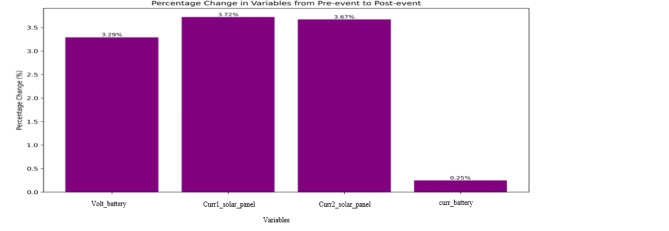
The mean of changes before and after the event for all parameters.

As shown in Fig. 7 Across all windows, the solar panel currents showed small but systematic increases. Average currents rose by + 3.72% (Panel 1) and + 3.67% (Panel 2) in the ± 4 day window. These results indicate that, while modest, the storm introduced measurable deviations that persist beyond the short-term spikes captured by CUSUM and z-scores.

The battery voltage displayed similarly small but systematic effects (d = 0.06–0.12), suggesting minor adjustments in power regulation during storm days. In contrast, battery current showed a window-length dependence: effects were visible at ± 2 days (d ≈ 0.07), weakened at ± 3 days, and vanished by ± 4 days (d ≈ 0.002, CI overlapping zero). This pattern highlights the transient nature of battery charging fluctuations, which diminish when averaged over longer periods, consistent with its buffering role.

Overall, the event-based approach demonstrates that the May 2024 storm did not merely produce isolated anomalies but also left a weak but systematic signature in solar panel and voltage telemetry when aggregated over several days. Importantly, all observed shifts remained well within the ± 10% design margin for MisrSat-2’s power subsystem, indicating no operational threat.

By providing a higher-level view of telemetry shifts, the event-based analysis bridges the gap between point anomalies (CUSUM, z-score) and system-wide behaviour, and it sets the stage for the subsequent validation and physical interpretation of these findings.

#### Integrated interpretation of detection results

A coherent pattern is evident across the analytical methods employed. CUSUM revealed clusters of step-like changes in solar panel currents on May 10, while z-scores confirmed these events by highlighting coincident current spikes. Event-based analysis further showed that the storm left small but systematic multi-day shifts in solar panel currents and battery voltage, though battery current effects quickly disappeared. Overall, the solar arrays registered modest storm-time anomalies, whereas the battery subsystem remained stable, buffering short-term fluctuations and preserving bus integrity.

As an alternative interpretation of the short-lived current excursions, we note that temperature-dependent photovoltaic I–V behaviour and local sensor/array heating may produce transient signatures in current and voltage independent of true changes in generated power; such temperature effects on space solar arrays^34^.

To corroborate this interpretation, we ran SPENVIS-EQUFLUX for the entire month of May 2024; the model predicts a month-integrated loss in solar-cell efficiency of ~ 0.32%, which we use as a qualitative benchmark for the radiation-driven change expected from the environment. Accordingly, the small, predicted degradation is aligned with the absence of any measurable radiation-driven signature in the telemetry, confirming that the observed variations remain below operational thresholds of concern.

### Validation of detected anomalies (testing significance)

As shown in Table 3, all candidate anomalies from CUSUM and z-score detection were subjected to statistical validation. Welch’s t-tests confirmed 14 events in Solar Panel 1 (SP1) and 16 events in Solar Panel 2 (SP1) (*p* < 0.05). Bootstrapping with 10,000 resamples further supported 12 (SP1) and 15 (SP2) of these events, reducing the likelihood that detections arose from sampling noise. To control for multiple testing, the Benjamini–Hochberg procedure (q = 0.05) retained 13 robust anomalies in SP1 and 17 in SP2, with the largest cluster occurring on May 10 between 07:05 and 09:25 UTC. By contrast, only two events were detected on May 11, neither of which passed all validation layers. The battery subsystem showed no confirmed anomalies under any test. MisrSat-2 experienced statistically robust solar panel disturbances primarily during the storm’s onset, while its power regulation system remained stable.

**Table 3:** Validation of detected anomalies in MisrSat-2 solar panel currents (May 9–15, 2024).

Validation method	Solar Panel 1	Solar Panel 2	Notes
CUSUM detections (raw)	29	30	All candidate change points identified (5-min cadence, illumination only)
Welch’s *t*-test (*p* < 0.05)	14	16	Initial statistical significance check
Bootstrapping (10,000 resamples)	12	15	Robust against small sample size
BH-FDR (q = 0.05)	13	17	Final robust anomalies retained after multiple-testing correction

Next, employ machine learning as a complementary layer to the statistical analysis, testing predictability from space-weather inputs and providing independent, interpretable confirmation of the detected patterns.

### Machine learning predictive results

While statistical analysis confirmed the presence and magnitude of storm-related anomalies, yet these remained descriptive, delineating the timing and locations of deviations. To investigate whether these disturbances could be anticipated from space weather drivers, we developed a MoE model for solar panel current prediction. The model was trained on May 2024 telemetry combined with geophysical indices and solar wind parameters, aiming at (i) accurately replicate nominal behaviour during quiescent conditions and (ii) detect deviations during storm periods as predictive anomalies.

#### Implementation Details

**Fig. 8:**
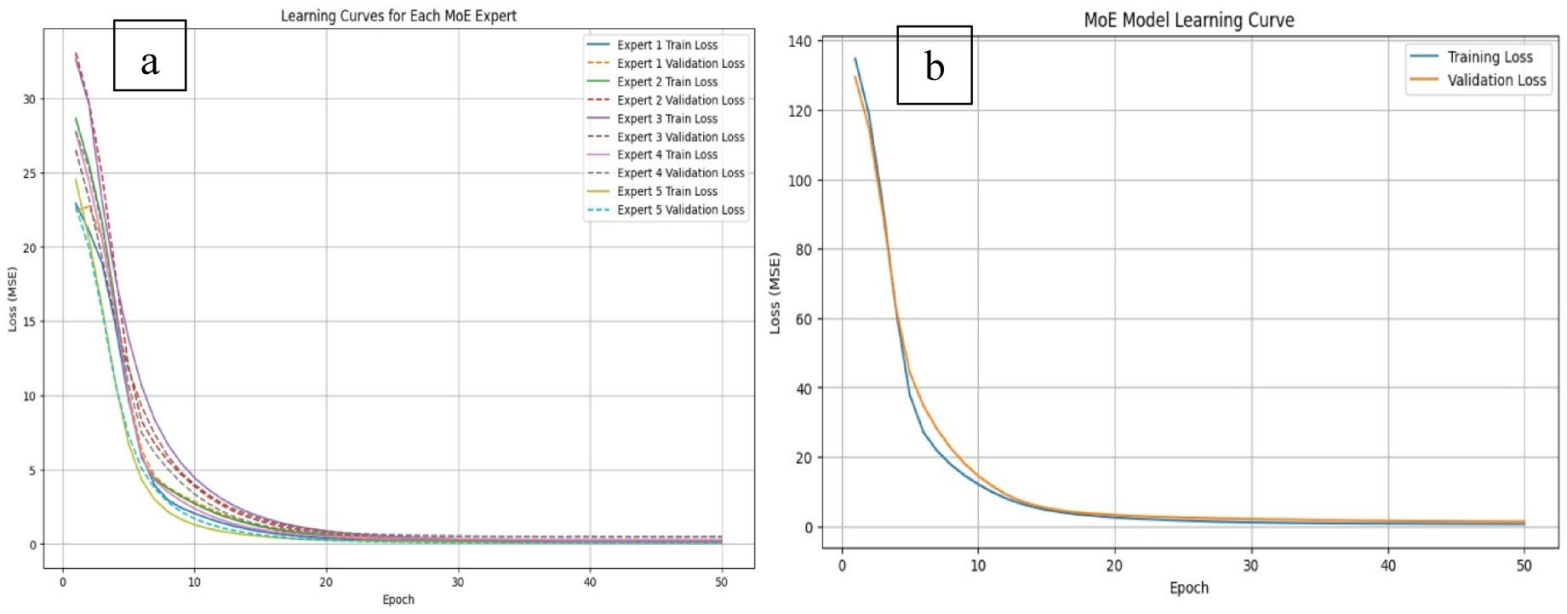
Learning curves for individual MoE experts and overall MoE model.

All models were implemented in Python using TensorFlow. An 80/20 train-test split was applied to assess generalization, following standard practice^26,35^. The 80/20 train–test split was applied in chronological order, ensuring that the model was trained on earlier data and evaluated on later unseen data, thereby preventing any temporal information leakage and ensuring a realistic assessment of generalization performance. Performance was evaluated using Mean Absolute Error (MAE) for average predictive deviation, Root Mean Squared Error (RMSE) to penalize larger errors^36,37^, and R^2^ to quantify explained variance^38^.Visual comparisons of predicted versus actual telemetry supported qualitative assessments, particularly around known storm events. The baseline models included Linear Regression, Random Forest, XGBoost, and Long Short-Term Memory (LSTM) networks. The LSTM model was implemented with a 24-time-step look-back window and 32 hidden units, followed by a dense output layer with a single unit. Training was performed using the Adam optimizer, and a dropout rate of 0.2 was applied to mitigate overfitting. Linear Regression served as a simple, interpretable benchmark^14^. Random Forest provided robust modeling of non-linear relationships with built-in feature importance^15^. XGBoost offered high accuracy and regularization for structured data^39^. LSTM was used to capture temporal dependencies and lagged effects^17^. To ensure the proposed MoE model generalization, learning curves for each expert as well as an overall MoE model learning curve are demonstrated in Fig. 8a, b. The learning curves for each expert show that both training and validation losses decrease steadily and converge to similar values after approximately 30 epochs, indicating no significant overfitting. Similarly, the overall MoE model learning curve exhibits a consistent reduction in both training and validation losses, with the validation loss stabilizing close to the training loss, further supporting the model’s ability to generalize.

Table 4 presents a comparative analysis of four models—Linear Regression, Random Forest, LSTM, XGBoost, and the proposed Mixture of Experts (MoE)—based on MAE, RMSE, and R^2^ metrics. The MoE model demonstrated superior performance, achieving the lowest MAE (0.063 A), RMSE (0.103 A), and the highest R^2^ (0.921), indicating strong predictive accuracy and variance capture. This is attributed to its specialized expert subnetworks and dynamic gating, enabling adaptability to complex input patterns, especially during space weather disturbances. LSTM followed, leveraging temporal dependencies to produce relatively low errors and a high R^2^ (0.881), though lacking MoE’s modular interpretability. Random Forest also performed well (R^2^: 0.854), effectively modeling nonlinear relationships but limited in temporal modeling. Linear Regression showed the weakest performance (R^2^: 0.742), reflecting the inadequacy of linear models for capturing complex satellite telemetry dynamics. Overall, MoE’s architecture proves most robust and adaptable for modeling nonlinear and variable-driven telemetry currents (Table 4).

**Table 4:** Performance comparison of baseline models and the proposed mixture of experts (MoE) model for telemetry current prediction.

Model	MAE (A)	RMSE (A)	R^2^ score
Linear regression	0.127	0.183	0.742
Random forest	0.091	0.145	0.854
LSTM (1 layer)	0.084	0.132	0.881
XGBoost	0.195	0.406	0.721
MoE (Proposed)	0.063	0.103	0.921

**Fig. 9:**
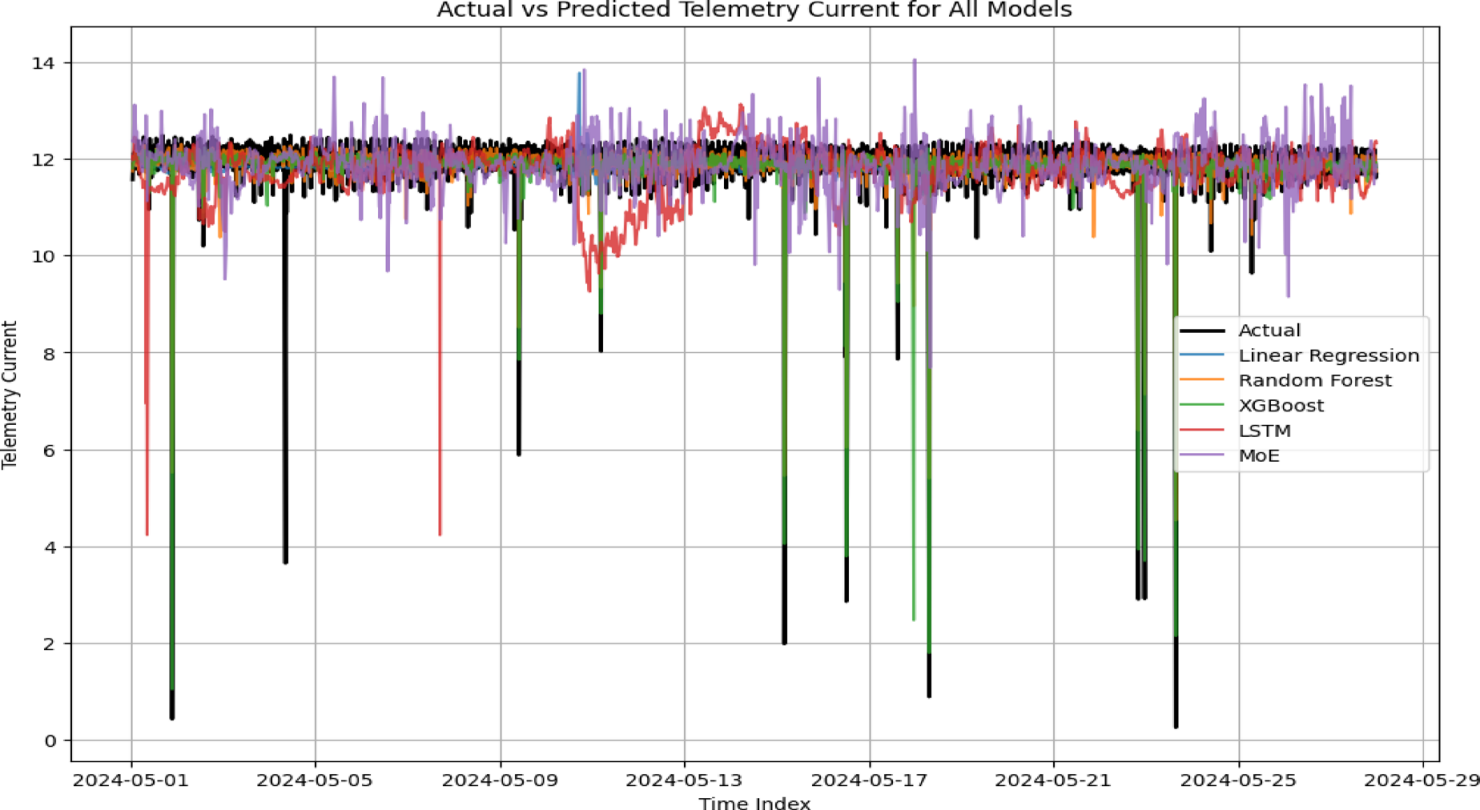
Actual versus predicted satellite telemetry current using multiple machine learning models**.**

Next**,** to gain insights into the internal behavior of the Mixture of Experts (MoE) model, we investigated both the temporal dynamics of expert selection and the feature specialization across experts. Figure 9 presents the daily expert activation heatmap for May 2024, highlighting the frequency with which each expert was selected as the dominant contributor (i.e., received the highest gating weight) per day. The results indicate clear temporal variation in expert engagement, reflecting the adaptive specialization characteristic of the MoE architecture. Expert 3 showed peak activation between May 11–13, a period associated with pronounced telemetry current fluctuations, suggesting its role in modeling anomalous or extreme conditions. In contrast, Experts 2 and 4 maintained steady activation, particularly in the latter half of the month, likely capturing regular operational dynamics under stable space weather conditions. Experts 0 and 1 were engaged more intermittently, possibly addressing rare or context-specific patterns. This dynamic allocation of sub-networks underscores the model’s ability to partition the input space into functionally distinct regimes. Such behaviour enhances both interpretability and predictive robustness compared to traditional monolithic architectures.

**Fig. 10:**
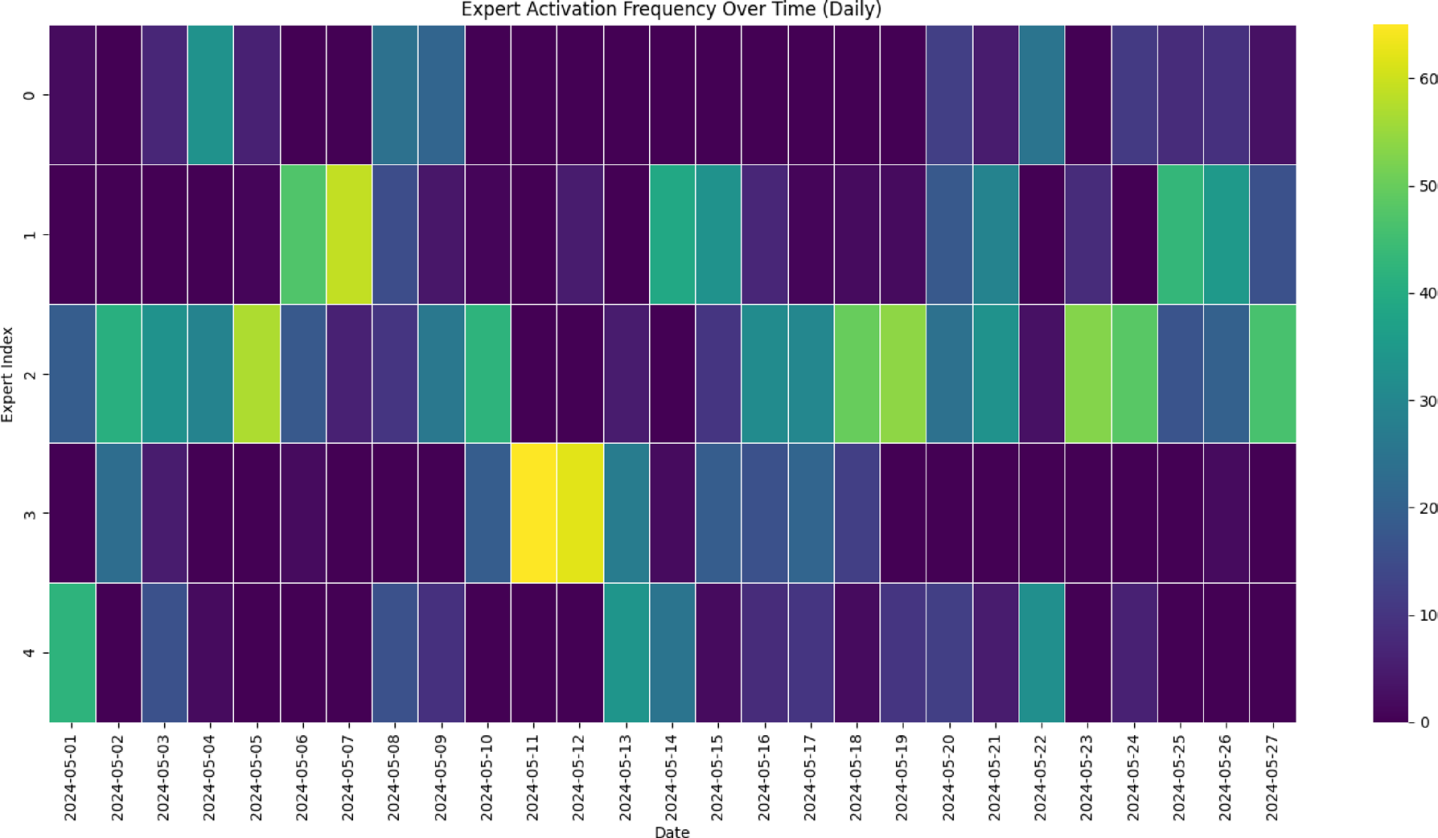
Daily activation frequency of MoE experts over May 2024.

**Fig. 11:**
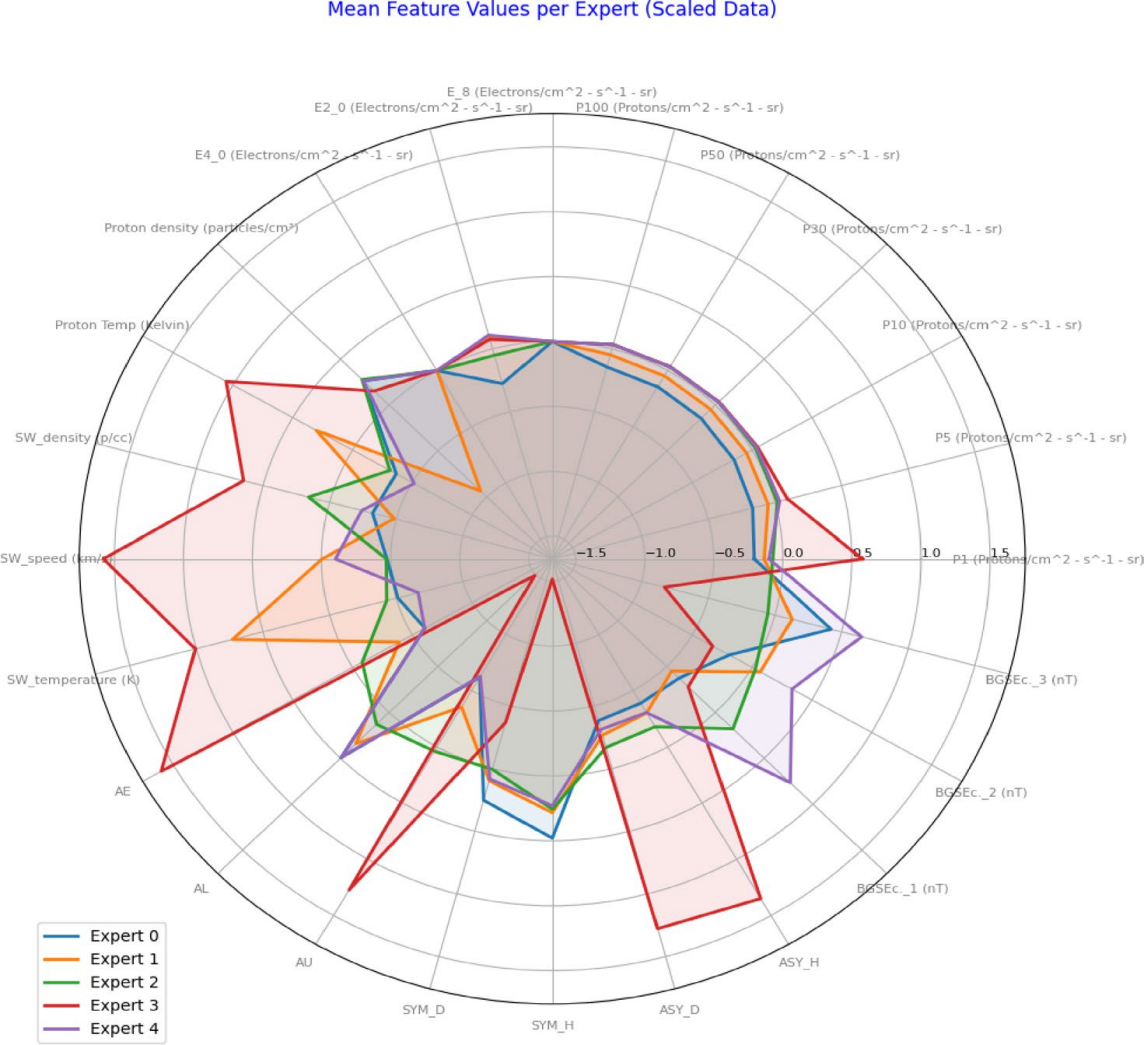
Radar plot showing mean scaled feature values per expert. Each line represents the average input profile handled by a specific expert in the MoE model. Expert 3 emphasizes disturbed space weather conditions (e.g., high solar wind speed and AE index), while others focus on more typical regimes, illustrating functional specialization among experts.

Figure 10 highlights temporal variation and specialization among experts. Expert 2 and Expert 3 are frequently active, while Expert 0 remains mostly inactive, indicating partial diversity but also imbalance in expert utilization. Complementing the temporal analysis, the radar plot in Fig. 11 summarizes the mean scaled feature values for the data segments assigned to each expert. This analysis provides a functional view of what kind of data conditions activate each expert. Expert 3 shows a pronounced preference for elevated values of solar wind speed, solar wind temperature, and geomagnetic indices (AE, AU, AL), suggesting its specialization in storm-time dynamics and high-energy space weather conditions. Expert 0 and Expert 1 show affinity for moderate values across most features, indicating a role in quiet-time operations or transition phases. Expert 2 and Expert 4 contribute more evenly across multiple features, reflecting possible generalist behavior or moderate sensitivity to a broader range of conditions. The presence of distinct and non-overlapping feature profiles across the five experts suggests that the MoE model has successfully learned to disentangle the multivariate input space and assign sub-models to specific operational regimes. This architecture inherently encourages diversity while minimizing redundancy, which is essential in high-dimensional, nonlinear systems such as satellite telemetry affected by space weather. Overall, these observations provide empirical evidence that the MoE routing mechanism not only aids in predictive accuracy (as shown in Table 4 and Fig. 9 but also offers interpretable pathways into the feature–response dynamics, a key advantage over traditional black-box models like LSTM.

#### Anomaly detection using Monte Carlo simulation

**Fig. 12:**
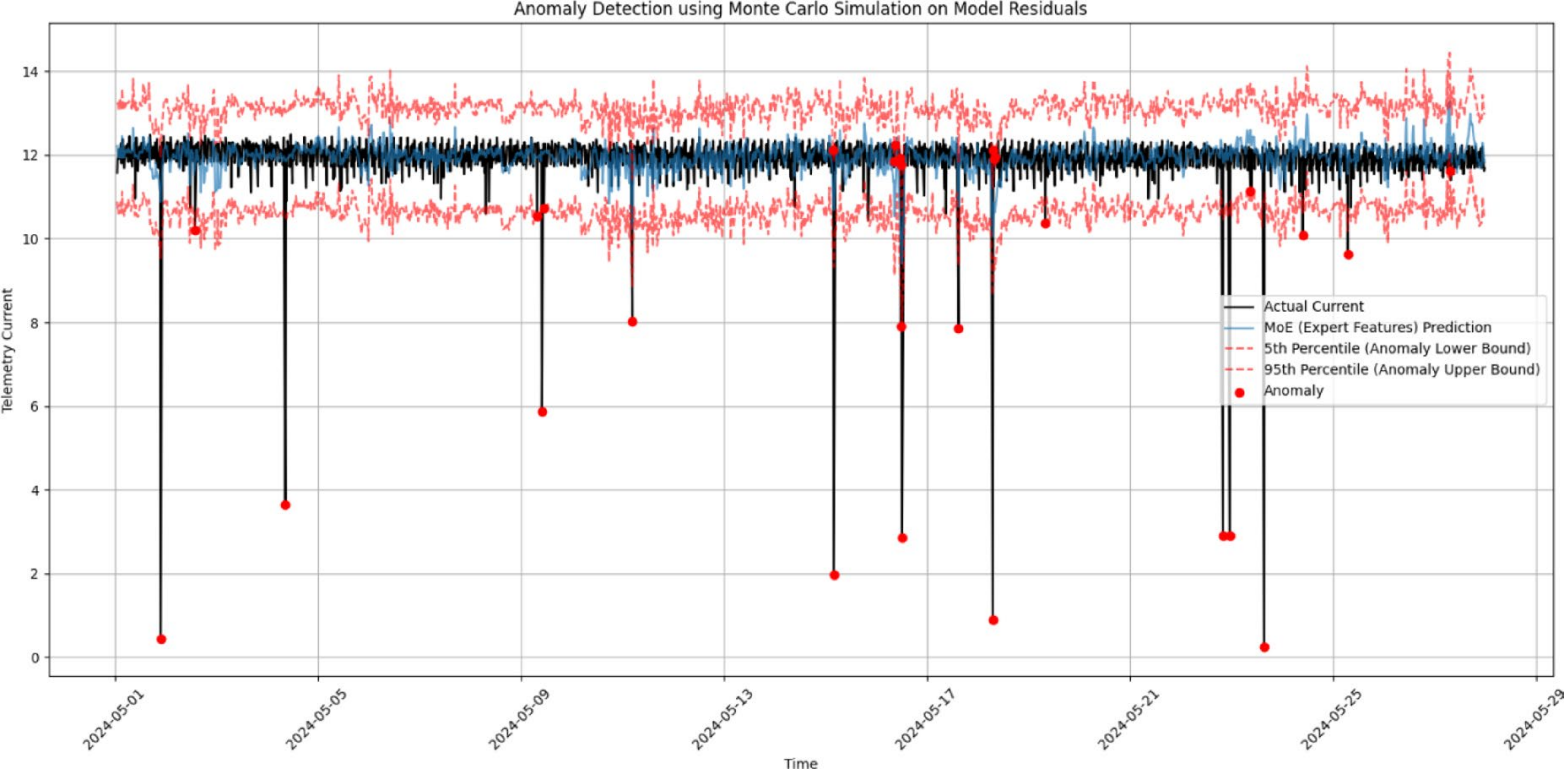
Anomaly detection using Monte Carlo simulation. The actual current (black) and MoE prediction (blue) are shown with 5th and 95th percentile bounds (red dashed). Red dots indicate detected anomalies beyond these bounds.

To evaluate the MoE model robustness in capturing abnormal telemetry behaviour under varying space weather conditions, Monte Carlo simulation was applied to its residuals for anomaly detection in satellite current data. Figure 12 illustrates the actual telemetry current compared to the predicted output of the MoE model, along with the 5th and 95th percentile confidence bounds generated from 1000 simulations of the residual distribution. Data points falling outside this range were flagged as anomalies and are highlighted in red. The majority of the predictions lie well within the probabilistic bounds, confirming the model’s general reliability under typical conditions. However, several distinct anomalies were observed, often corresponding to sharp drops in measured current. These anomalies may indicate either transient space weather disturbances or operational shifts in satellite orientation, eclipse entry, or sensor limitations. Similar anomaly detection methods have proven effective in satellite telemetry systems, especially when leveraging probabilistic thresholds rather than fixed rules, as highlighted in prior studies^37^.

**Fig. 13:**
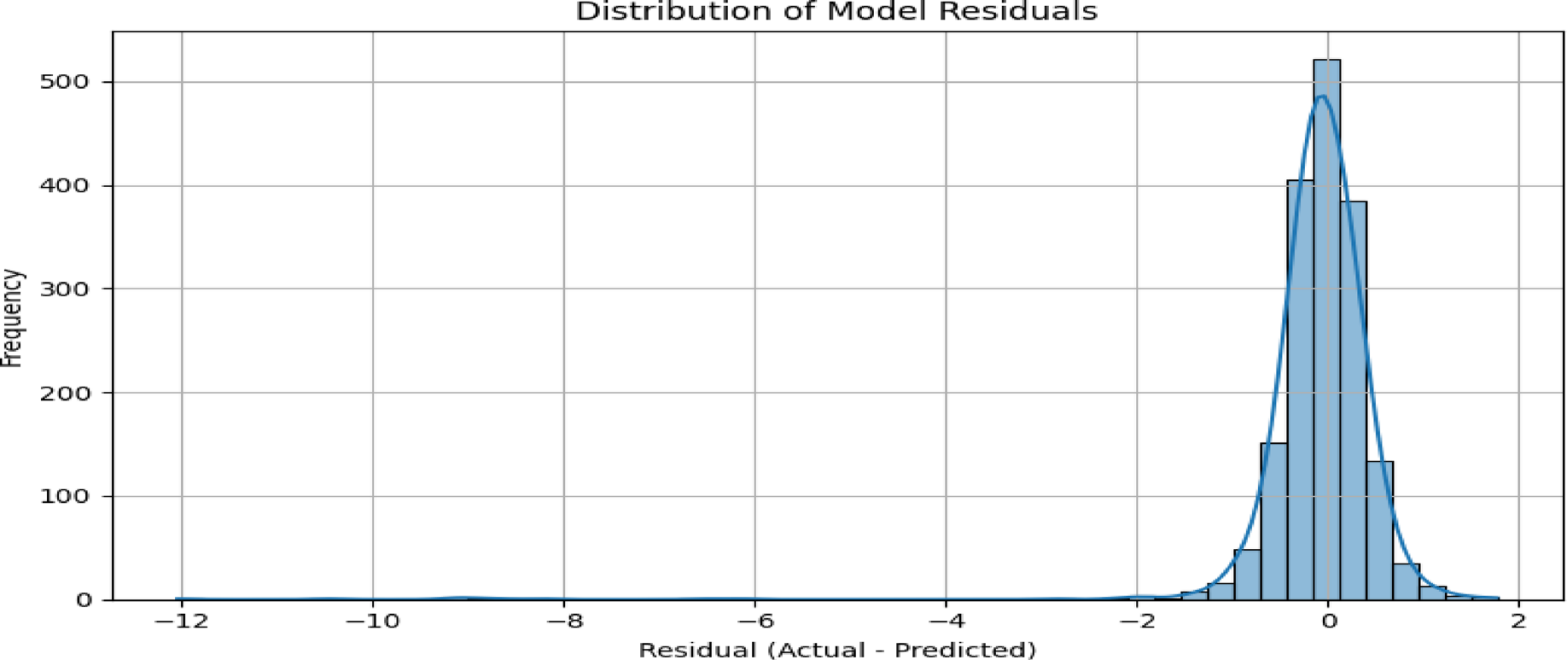
Residual distribution from the MoE model. The histogram shows a near-normal shape with a slight negative skew, used for generating anomaly thresholds.

To validate the statistical soundness of this simulation-based approach, we examined the residual distribution (Fig. 13). The residuals exhibit a near-normal distribution centered around zero, with a modest negative skew and a heavy tail on the left. This indicates that the model slightly underestimates telemetry current during some high-deviation events—an effect that may stem from unmodeled nonlinearities in the space weather influence or signal loss during eclipse or plasma events. Despite this skew, the tight concentration of most residuals around zero reflects a high degree of prediction accuracy under nominal conditions.

Overall, the Monte Carlo anomaly detection method effectively complements deterministic modeling by quantifying uncertainty and highlighting statistically significant deviations. This dual approach improves confidence in operational forecasting and anomaly attribution, offering a more comprehensive framework for health monitoring of spacecraft power systems.

This study demonstrates the effectiveness of advanced machine learning approaches, particularly the Mixture of Experts (MoE) architecture, in modeling and interpreting satellite telemetry data under varying space weather conditions. Among all evaluated models, the MoE framework achieved superior predictive accuracy, as evidenced by the lowest MAE and RMSE and the highest R^2^ values (Table 4), outperforming both traditional (Linear Regression, Random Forest) and neural based (LSTM) baselines. The success of the MoE model stems from its modular design, which enables adaptive routing of inputs to be specialized sub-networks, allowing it to capture complex, nonlinear relationships across distinct operational regimes.

Temporal analysis of expert activation revealed functional specialization among experts, with Expert 3 consistently selected during geomagnetically disturbed periods. This aligns with the radar plot findings, which showed that Expert 3 responds to high-energy solar wind conditions and elevated geomagnetic indices. Such specialization suggests that the MoE architecture effectively decomposes the multivariate input space and routes inputs to the most appropriate sub-models, enhancing both accuracy and interpretability. Complementary to the modeling results, event-based analysis during the May 2024 geomagnetic storm highlighted statistically significant current fluctuations in the solar panels, especially during storm onset on May 10–11. While these variations did not propagate to the battery system—indicating effective power regulation—their alignment with space weather parameters supports the role of environmental disturbances in transient telemetry anomalies.

Finally, the application of Monte Carlo simulations to MoE residuals provided a robust mechanism for anomaly detection. By establishing probabilistic bounds around model predictions, this approach identified statistically significant deviations potentially linked to space weather or operational transitions. Overall, the integration of MoE modeling with event detection and probabilistic analysis offers a comprehensive, interpretable, and operationally relevant framework for satellite power system monitoring under dynamic environmental conditions.

## Conclusion

This study investigates the impact of the May 2024 geomagnetic storm on MisrSat-2, an Earth observation satellite launched in December 2023 without onboard space weather sensors. To address this limitation, high-resolution telemetry was integrated with solar wind and geomagnetic data to assess system responses. A hybrid framework—combining statistical analysis, machine learning model, and radiation damage simulation—was developed to evaluate storm-induced effects on solar panel current and battery telemetry.

The statistical pipeline—comprises of CUSUM change-point detection, z-score outlier identification, and event-based analysis—revealed modest storm-time variations in solar panel currents on May 10, coinciding with the storm’s onset. Rigorous validation through Welch’s t-tests, bootstrapping, false discovery rate control, and effect size analysis confirmed that these deviations were small (< 4%) and remained within engineering design tolerances. In contrast, the battery subsystem showed stable current–voltage behaviour, reflecting its role as a buffer against short-term fluctuations. The SPENVIS-based degradation analysis confirmed minimal radiation-induced damage (~ 0.32%), consistent with the short operational duration of the satellite and its use of triple-junction GaAs solar cells.

Among the tested predictive models, the MoE framework exhibited superior performance (MAE = 0.063 A, R^2^ = 0.921), surpassing LSTM, Random Forest, XGBoost, and Linear Regression baselines. Its architecture, which enables functional specialization across space weather regimes, proved particularly effective in capturing complex, nonlinear interactions during storm-time conditions. Visualization of expert activation patterns and SHAP-based feature importance highlighted the model’s capacity for interpretable forecasting and adaptive response to input variations.

Overall, MisrSat-2 demonstrated resilience to the May 2024 geomagnetic storm, with all detected effects remaining well within safe margins. The integration of statistical, physical, and machine learning approaches presented here offers a scalable methodology for anomaly diagnostics and mission assurance, particularly for small satellites without onboard radiation monitors. Future work should extend this framework to multi-satellite constellations, incorporate longer temporal baselines, and explore causal inference and physics-informed neural architectures to further enhance robustness and attribution capability in operational settings.

## Limitations and future work

This study has several limitations that should be acknowledged. First, the analysis relied solely on MisrSat-2 telemetry, which does not include dedicated radiation or plasma sensors. As a result, storm impacts had to be inferred indirectly from power subsystem behaviour. Access to complementary data from other satellites equipped with particle and field detectors would provide a stronger basis for attribution.

Second, while the machine learning models demonstrated predictive skill, they were trained on a relatively short, storm-centered dataset and may not fully generalize to other conditions or platforms. The Mixture of Experts model effectively captured regime shifts but remains limited in interpretability and may not resolve causal pathways between drivers and responses without additional constraints.

Future work should address these limitations by integrating multi-satellite datasets to better capture environmental drivers and by extending the training set across multiple storms and operational contexts to improve model robustness. Advanced machine learning techniques, such as Mixture of Experts architectures based on ensemble autoencoders, could enhance anomaly classification and regime separation. In addition, emerging data-driven causal inference frameworks (Runge et al. 2024) offer a systematic means of distinguishing between competing explanations and strengthening causal attribution^40^. Together, these approaches will improve the interpretability of automated anomaly detection and help differentiate between anomalies induced by space weather and those arising from normal operational dynamics.

Despite these constraints, the present framework demonstrates practical utility for on-orbit anomaly monitoring, providing a scalable and interpretable approach to satellite health assessment under extreme space weather conditions.

## Data and materials availability

The data used in this study are available from public databases and the Egyptian Space Agency upon reasonable request. The datasets generated and/or analyzed during the current study are available from the corresponding author, [Dalia Elfiky], upon reasonable request (email: [delfiky@narss.sci.eg]).

## Data Availability

The code used for analysis is **publicly available at** https://github.com/Hamada224/MisrSat2-Reproducibility.git.
